# Elevated expression of the toll like receptors 2 and 4 in obese individuals: its significance for obesity-induced inflammation

**DOI:** 10.1186/1476-9255-9-48

**Published:** 2012-11-28

**Authors:** Rasheed Ahmad, Anfal Al-Mass, Valerie Atizado, Asma Al-Hubail, Fahad Al-Ghimlas, Monira Al-Arouj, Abdullah Bennakhi, Said Dermime, Kazem Behbehani

**Affiliations:** 1 Immunology and Cell Therapy Unit, Department of Biomedical Research, Dasman Diabetes Institute, Kuwait, P.O. Box 1180, Dasman 15462, Kuwait; 2 Tissue Bank Core Facility, Dasman Diabetes Institute, Dasman, Kuwait; 3 Clinical Services-clinical laboratory, Dasman Diabetes Institute, Dasman, Kuwait; 4 Fitness and Rehabilitation Center, Dasman Diabetes Institute, Dasman, Kuwait; 5 Senior Managements, Dasman Diabetes Institute, Dasman, Kuwait

## Abstract

**Background:**

Expression profile of the toll like receptors (TLRs) on PBMCs is central to the regulation of proinflammatory markers. An imbalance in the TLRs expression may lead to several types of inflammatory disorders. Furthermore, the dynamic regulation of inflammatory activity and associated impaired production of cytokines by peripheral blood mononuclear cells (PBMCs) in obese individulas remain poorly understood. Therefore, we determined the perturbation in TLRs (TLR2 and TLR4), their adaptor proteins (MyD88, IRAK1 and TRAF6) expression in PBMCs/subcutaneous adipose tissue (AT) as well as inflammatory cytokines changes in obese individuals.

**Methods:**

mRNA expression levels of TLR2, TLR4, IL-6, TNF-α and adaptor proteins were determined by RT-PCR. TLR2, TLR4 and adaptor proteins expression in AT was determined by immunohistochemistry.

**Results:**

Obese and overweight individuals showed significantly increased expression of TLR2, TLR4 and MyD88 in both PBMCs and AT as compared with lean individuals (P < 0.05). Interestingly, we found a remarkably higher expression of TLRs in obese and overweight individuals with type 2 diabetes (P < 0.05). Increased expression of TLR2, TLR4, MyD88 and IRAK1 correlated with body mass index (BMI) (TLR2: r = 0.91; TLR4: r = 0.88, P <0.0001; MyD88: r = 0.95, P < 0.0001; IRAK1 r = 0.78, P < 0.002). TLRs’ expression was also correlated with fasting blood glucose (FBG) (TLR2: r = 0.61, P < 0.002; TLR4: r = 0.52, P < 0.01) and glycated haemoglobin (HbA1c) ( TLR2: r = 0.44, P <0.03; TLR4: r = 0.48, P < 0.03). Transcript levels of IL-6 and TNF-α were highly elevated in obese subjects compared to lean subjects. There was a strong association of TLRs’ expression in PBMCs with TNF-α (TLR2: r = 0.92; TLR4: r = 0.92; P < 0.0001) and IL-6 (TLR2: r = 0.91, P < 0.0001; TLR4: r = 0.81; P < 0.001). Similarly adaptor proteins were significantly correlated with TNF-α (MyD88: r = 0.9, P < 0.0001; IRAK1: r = 0.86; P < 0.0002) and IL-6 (MyD88: r = 0.91, P < 0.0001; IRAK1: 0.77; P < 0.002).

**Conclusions:**

TLRs and adapter proteins were overexpressed in PBMCs from obese subjects, which correlated with increased expression of TNF-α and IL-6. This association may explain a potential pathophysiological link between obesity and inflammation leading to insulin resistance.

## Introduction

Obesity is associated with a low-grade systemic chronic inflammation that is linked to insulin resistance, cardiovascular diseases and type-2 diabetes [1-3]. Obesity-induced inflammation is characterized by the abnormal production of pro- and anti-inflammatory adipocytokines. It has been found that resident macrophages in adipose tissue are mainly responsible for the production of inflammatory cytokines. The dynamic regulation of inflammatory activity and associated impaired production of cytokines/chemokines by PBMCs and adipocytes from obese individuals remain poorly understood. Changes in the expression profile of different receptors, ligands and adhesion molecules on PBMCs may lead to the development of different immunological diseases.

TLRs are transmembrane proteins involved in detection of microbes upon infection and play a crucial role in the host immune defence; 13 members of the TLR family in mammals (11 members in human) are known so far [4]. Structurally, TLRs are characterized by an extracellular leucine-rich repeat (LRR) domain, a domain involved in the recognition of pathogen-associated molecular patterns (PAMPs) and a cytoplasmic Toll/IL-1 (TIR) domain that activates downstream signaling molecules including MyD88, IRAKs and TRAF6 [5]. Activation of these adaptor proteins stimulates multiple cascades including extracellular signal-regulated kinase (ERK), c-Jun N-terminal kinase (JNK) and p38 mitogen-activated protein kinases (MAPK) pathways and activation of NF-kB signaling and the resulting up-regulation of diverse inflammatory mediators, such as cytokines, chemokines, and adhesion molecules, which together, serve essential functions in promoting inflammation. Increased activity of TLR2 and TLR4 has been found in diabetic patients and is associated with the pathogenesis of diabetes and atherosclerosis in both clinical and experimental conditions [6-9]. TLR2 and TLR4 bind to components of the Gram-positive and -negative bacteria, respectively [10]. In addition, ligands for TLR2 and TLR4 include free fatty acids, high-mobility group B1 protein (HMGB1), heat shock protein-60 (HSP60), heat shock protein-70 (HSP70), endotoxin, hyaluronan, advanced glycation end (AGE) products, and extracellular matrix components [10].

The role of TLR2 and TLR4 has been suggested in conventional insulin resistance (IR) target tissues like skeletal muscle and adipose tissue of Type 2 diabetes subjects [11,12]. TLR4 was described as a molecular link between free fatty acids, inflammation, and the innate immune system [6]. High TLR4 mRNA expression was reported in differentiating adipose tissue of *db*/*db* mice [13]. Adipocytes produced IL-6 via TLR4 activation and the upregulation of osteopontin further exacerbated adipose tissue inflammation and insulin resistance [14]. While these important observations from animal and human tissue data create an interest to see a role for TLR2 and TLR4 in obesity, it remains unknown whether alterations in TLR2 and TLR4 expression and associated inflammatory cytokines contribute to systemic inflammation which causes induction of insulin resistance in obese individuals. Therefore, we determined the changes in expression of TLRs in PBMCs/AT and associated inflammatory cytokines (TNF-α and IL-6) in obese individuals. We found elevated expression of TLRs and their adaptor proteins in PBMCs and AT. Elevated expression of TLRs and their adaptor proteins were significantly correlated with inflammatory cytokines. The localization of TLRs in adipose tissue was confirmed by immunohistochemistry. This study supports a model where the increased TLRs’ expression relates with the elevated cytokine expression in PBMCs from obese subjects.

## Methods

### Study Participants and clinical laboratory evaluation

43 individuals were recruited from the local clinics. Written informed consent was obtained from all participants for inclusion in the study and the study protocol was approved by the institutional ethics committee (Ethical Review Committee of Dasman Diabetes Institute). Lean, overweight and obese subjects are asymptomatic or free of disease. Diabetic subjects were on medication of glucophage alone or in combination with crestor or zestril or januvia. Blood samples were collected after overnight fasting for isolation of PBMCs and for the determination of blood-derived factors including blood glucose, total cholesterol, high density lipoprotein cholesterol (HDL-C), low-density lipoprotein cholesterol (LDL-C), and hemoglobin A1c (HbA1c). Participants’ age, height, weight, blood pressure and diabetes status were assessed at the time of the blood draw. All biochemical tests were performed by using standard kits. The characteristics of the participants are described in Table 1. Adipose tissue samples were obtained from 21 individuals with different BMI.

**Table 1:** Characteristics of the study participants

**Characteristic**	**Lean**		**Overweight**		**Obese**	
	**Non**-**Diabetic**	**Diabetic**	**Non**-**Diabetic**	**Diabetic**	**Non**-**Diabetic**	**Diabetic**
Age range (years)	25-48	41-53	28-52	42-63	29-57	27-58
Body mass index	23.19 ± 1.27	23.71 ± 0.97	28.23 ± 1.16	27.99 ± 0.71	34.71 ± 2.97	32.73 ± 2.45
Fasting blood glucose (mmol/l)	5.00 ± 0.77	9.40 ± 5.37	5.04 ± 0.47	9.46 ± 2.55	5.73 ± 0.95	9.70 ± 3.04
Glycosylated haemoglobin (%)	5.73 ± 0.98	9.85 ± 1.90	5.52 ± 0.56	9.35 ± 2.73	5.84 ± 0.65	8.12 ± 1.61
Cholesterol (mmol/l)	4.85 ± 0.94	4.70 ± 15	4.80 ± 0.73	4.61 ± 0.57	5.33 ± 1.06	5.71 ± 0.90
HDL cholesterol (mmol/l)	1.27 ± 0.31	1.17 ± 0.41	1.18 ± 0.17	1.40 ± 0.47	1.13 ± 0.24	1.07 ± 0.13
LDL cholesterol (mmol/l)	3.05 ± 0.81	2.95 ± 1.48	3.10 ± 0.68	2.52 ± 0.22	3.80 ± 2.25	3.25 ± 0.70
Triglycerides (mmol/l)	1.12 ± 0.67	1.24 ± 0.95	1.14 ± 0.72	1.48 ± 0.99	1.97 ± 1.13	2.95 ± 1.12

### Peripheral blood mononuclear cells (PBMCs)

For PBMCs isolation, fresh blood samples were collected from participants in EDTA-tubes. PBMCs were isolated by using Ficoll-Hypaque density gradient centrifugation [15].

### Subcutaneous adipose tissue biopsy

Human adipose tissue samples were collected via abdominal subcutaneous fat pad biopsy lateral to the umbilicus using standard surgical techniques. In brief, the region was sterilized using alcohol, and locally anesthetized with 2 ml 2% lidocaine. Through a small superficial 0.5 cm skin incision, fat tissue was collected via punch biopsy. Tissue samples were stored in formalin or snap-frozen in liquid nitrogen and stored at −80°C. Samples were then used for immunohistochemical staining.

### Immunohistochemistry

Frozen sections (4 μm) of adipose tissue were cut. Slides were deparaffinized in xylene and rehydrated in pure ethanol to water. Antigen retrieval was done by placing the slides in target retrieval solution pH6.0 (Dako) in the pressure cooker boiling for 8 minutes and cooling down for 15 minutes. After washing in phosphate buffer saline (PBS), endogenous peroxidase was blocked with 3% H_2_O_2_ for 30 minutes. The slides were blocked with 5% milk for 1 hour followed by the 1 hour incubation with 1%BSA solution. The slides were incubated in primary antibody overnight at room temperature in 1:300 dilution of rabbit polyclonal TLR-2 antibody (ProSci Incorporated), 1:400 dilution of rabbit polyclonal TLR-4 antibody (ProSci Incorporated), 1:200 dilutions of rabbit polyclonal antibody (ProSci Incorporated), 1:200 dilution of TRAF6 antibody (ProSci Incorporated), 1:200 dilution of MyD88 (ProSci Incorporated) and 1:200 dilution of IRAK antibody (ProSci Incorporated). After washing with PBS–0.05% Tween, slides were incubated for 1 hour with the respective secondary antibodies. Bound antibody was detected with HRP EnVision Plus Kit. Color was developed in 3, 3’-diaminobenzidine chromogen substrate. The sections were then washed in running tap water, lightly counterstained with Gill’s hematoxylin, dehydrated through ascending graded alcohols, cleared in xylene, and mounted in DPX. Two different observers who were blinded to the source of the tissues quantified the expression of each antigen semiquantitatively on a 3-point scale, where 0 = no staining; 1 = mild expression, limited areas stained; and 3 = strong expression, strong overall staining. Immunohistochemical staining of the section of human spleen tissue (HST) was used as positive control for TLR2. Immunohistochemical staining of the section of breast cancer tissue was used as positive control for TLR4 [16].

### Reverse Transcription Polymerase Chain Reaction

Total RNA was extracted using RNeasy kit (Qiagen). The cDNA was synthesized with 0.5ug of total RNA using high capacity cDNA reverse transcription kit (Invitrogen). Polymerase chain reaction (PCR) was performed using the One Step PCR kit (Promega). The primer pairs used were as follows: TLR2 Fwd (5′-ATTGTGCCCATTGCTCTTTC-3′) and TLR2 Rev (5′-TTCTTCCTTGGAGAGGCTGA −3′); TLR4 Fwd (5′-AATCCCCTGAGGCATTTAGG-3′) and TLR4 Rev (5′-CCCCATCTTCAATTGTCTGG −3′); MyD88 Fwd (5′-GGATGGTGGTGGTTGTCTCT-3′) and MyD88 Rev (5′-AGGATGCTGGGGAACTCTTT-3′); IRAK1 Fwd (5′- AGCCCCTTCTTCTACCAAGC-3′) and IRAK1 Rev (5′-AGGAAGCTCTGCTTCACTGC-3′) TRAF6 Fwd (5′-CTGCAAAGCCTGCATCATAA-3′) and TRAF6 Rev (5′- GGGGACAATCCATAAGAGCA −3′); TNF-α Fwd (5′-CAGAGGGCCTGTACCTCATC-3′) and TNF-α Rev (5′- GGAAGACCCCTCCCAGATAG −3′); IL-6 Fwd (5′- CAGGGGTGGTTATTGCATCT-3′) and IL-6 Rev (5′-AAAGAGGCACTGGCAGAAAA-3′) and GAPDH Fwd (5′-ATCGTGGAAGGACTCATGACCACA-3′) and GAPDH Rev 5′-TAGAGGCAGGGATGATGTTCTGGA-3′. The RNA was reverse-transcribed at 50°C for 30 minutes and reverse transcriptase was inactivated at 95°C for 15 minutes. PCR was run at: 30 cycles of 94°C for 1 minute, 50°C for 1 minute, 72°C for 1 minute, and a final extension step at 72°C for 10 minutes. The PCR product (10 μl) was analyzed on 1.4% agarose gel to detect TLR2, TLR4, MyD88, IRAK1, TRAF6, IL-6, TNF-α and GAPDH cDNA amplification. Relative band quantification was performed by using Gel Doc™ XR + imaging system (Bio Rad,USA). Density of the bands was expressed in arbitrary units. Jurkat cells were used as a –ve control for TLR2 and TLR4 [17]. THP1 cells were used for + ve control for TLR2 and TLR4 [18].

### Statistical analysis

Data were presented as mean ± standard deviation, unless otherwise indicated. Unpaired Student t test was used to compare means between groups. Correlation and linear regression were used to see the association between different variables. For all analyses, *P* value < 0.05 was considered significant. All statistical analysis was performed with GraphPad Prism software (La Jolla, CA, USA).

## Results

### Elevated expression of TLRs in PBMCs from obese subjects

**Figure 1:**
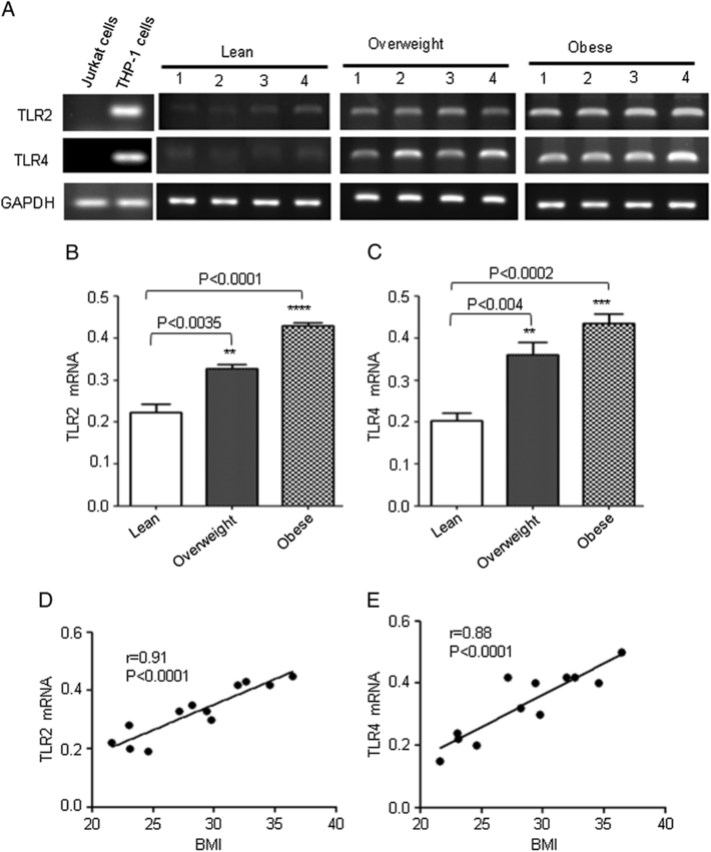
**Expression of TLR2 and TLR4 mRNA in PBMCs. **The PBMCs from 4 individuals in each group (lean, overweight and obese) were used for total RNA isolation. TLR2 and TLR4 mRNA were measured by RT-PCR. Jurkat cells do not express TLR2 and TLR4 (−ve control). THP1 cells express TLR2 and TLR4 (+ve control) (**A**). The RNA expression was normalized to GAPDH housekeeping gene expression. TLR2 and TLR4 mRNA expression is shown in arbitrary units for each group. Results are expressed relative to the expression level in controls and are represented as means ± SE values (**B** and **C**). A strong association of TLR2/ TLR4 mRNA with BMI was observed in PBMCs (**D** and **E**).

Peripheral blood mononuclear cells (PBMCs) are often considered for investigating many aspects of immunological responses. In previous studies, it has been shown that TLR2 and 4 are highly expressed in PBMCs during course of inflammatory disorders. However, since expression of TLR2 and 4 in PBMCs in obesity has yet not been well defined, we measured levels of mRNA for TLR2 and 4 in PBMCs from obese, overweight and lean individuals. As shown in Figure 1A, TLR2 mRNA showed the highest expression, followed by TLR4 in obese subjects and there was significant difference from lean subjects (P < 0.05). Significant differences in the expression of TLRs were also found between overweight and lean subjects (P < 0.05) (Figure 1B, C). Expression levels of TLRs increased significantly with increasing BMI (TLR2: r = 0.91; TLR4: r = 0.88, P < 0.0001) (Figure 1D, E).

### Elevated expression of adaptor proteins in PBMCs from obese subjects

**Figure 2:**
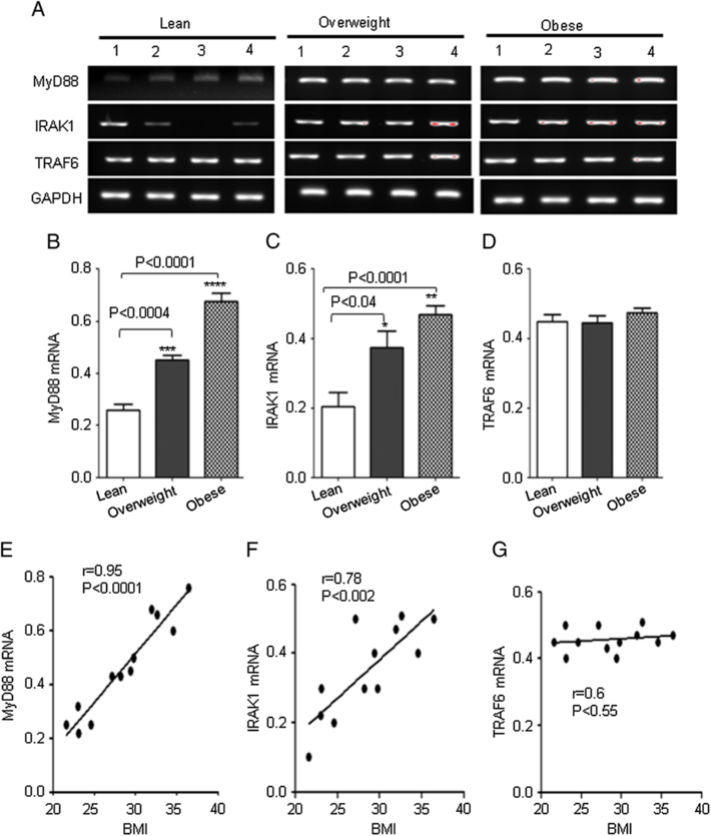
**Expression of TLRs’ adaptor proteins in PBMCs. **The PBMCs from 4 individuals in each group (lean, overweight and obese) were used for total RNA isolation. MyD88, IRAK1 and TRAF6 mRNA were measured by RT-PCR (**A**). The RNA expression was normalized to GAPDH housekeeping gene expression. MyD88, IRAK1 and TRAF6 mRNA expression is shown in arbitrary units for each group. Results are expressed relative to the expression level in controls and are represented as means ± SE values (**B**, **C** and **D**). A strong association of MyD88 and IRAK1 with BMI was observed in PBMCs (**E** and **F**).

TLRs’ activation requires the signal transduction molecules myeloid differentiation protein (MyD88), IL-1 receptor-associated kinase 1(IRAK1), and tumor necrosis factor (TNF) receptor-associated kinase 6 (TRAF6) which led to the hypothesis that these TLR’s adaptor proteins (MyD88, IRAK1 and TRAF6) could be increased in obese individuals. Therefore, we also measured the expression of TLRs’ adaptor proteins MyD88, IRAK1 and TRAF6 in PBMCs used in the previous experiment. We found that expression levels of MyD88 and IRAK1 were significantly increased in PBMCs from overweight and obese subjects (P < 0.05) (Figure 2A, B, C). However we noticed a strong constitutive expression of TRAF6 in PBMCs from all groups of subjects (Figure 2A, D). Linear regression analysis revealed a significantly positive correlation between MyD88/IRAK1 expression and BMI (MyD88: r = 0.95, P < 0.0001; IRAK1: r = 0.78, p < 0.002) (Figure 2E, F). No association was observed between TRAF6 expression and BMI (Figure 2G).

### TLRs and MyD88 are upregulated in subcutaneous adipose tissue from obese subjects

**Figure 3:**
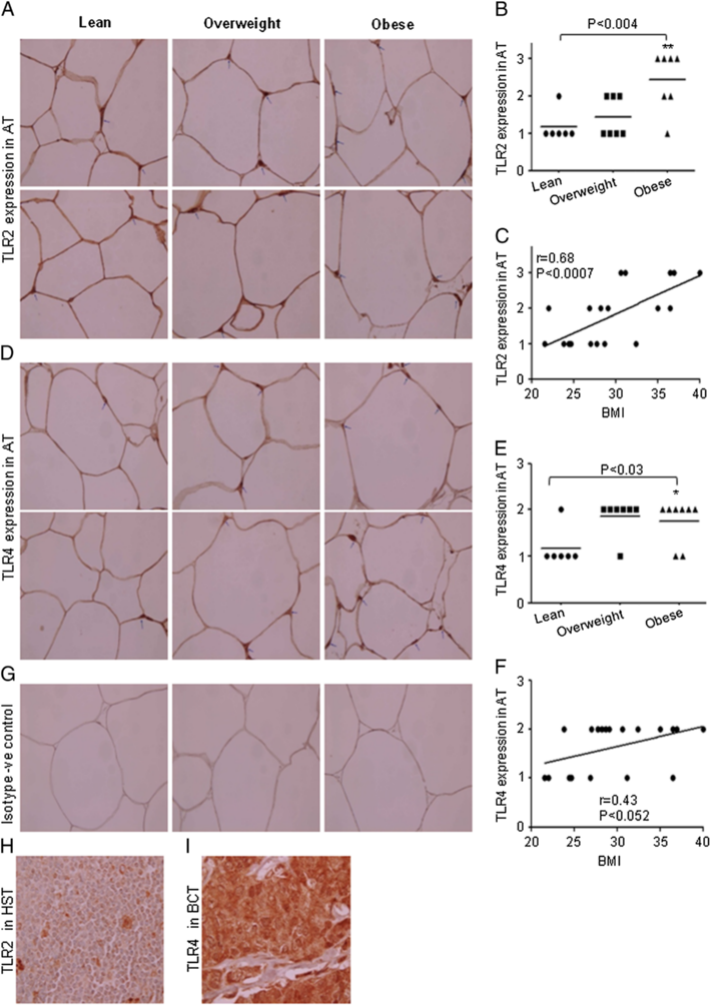
**Expression of TLR2 and TLR4 in adipose tissues. **Sections of the adipose tissues from individuals (Obese 8; Overweight 7 and 6 Lean) were stained with TLR2, TLR4 or with control antibodies . All sections were counterstained with hematoxylin. Positive signals appear in red (original magnification X100). Two representative sections of the adipose tissues from each group were shown. Arrowheads indicate intensity of the expression of TLR2 and TLR4 on the cells (**A** &**D**). Intensity of expression was assessed and an expression score was assigned according to following scale: 0 (no staining) and 3 (strong staining). Horizontal bars show the mean (**B** &**E**). Correlation and simple linear regression of AT expression of TLR2 and TLR4 with BMI was observed (**C **and **F**). Isotype –ve control (**G**). Section of human spleen tissue (HST); positive control for TLR2 (**H**). Section of breast cancer tissue (BCT); positive control for TLR4 (**I**).

**Figure 4:**
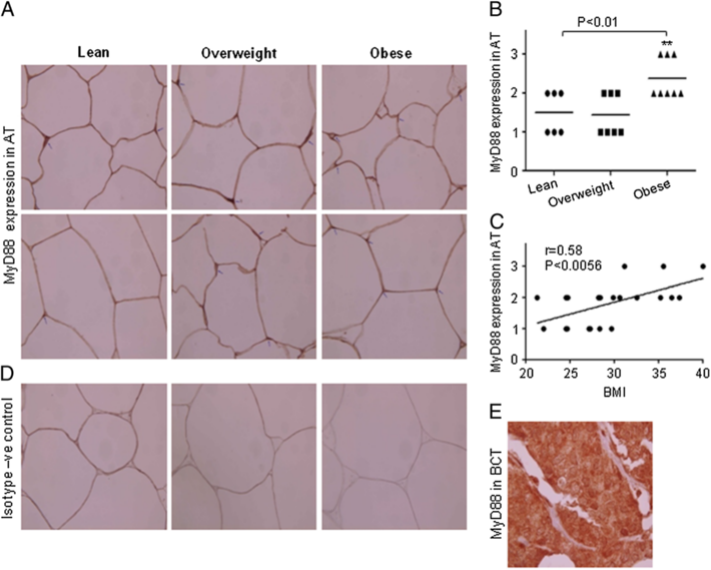
**Expression of MyD88 in adipose tissues. **Sections of the adipose tissues from individuals (Obese 8; Overweight 7 and 6 Lean) were stained with MyD88 or with control antibodies . All sections were counterstained with hematoxylin. Positive signals appear in red (original magnification X100). Two representative sections of the adipose tissues from each group were shown. Arrowheads indicate intensity of the expression of MyD88 on the cells (**A**). Intensity of expression was assessed and an expression score was assigned according to following scale: 0 (no staining) and 3 (strong staining). Horizontal bars show the mean (**B**). Correlation and simple linear regression of AT expression of MyD88 with BMI was observed (**C**). Isotype –ve control (**D**). Section of BCT; positive control for MyD88 (**E**).

To identify TLR2 and TLR4 expression in adipose tissues, we performed immunohistochemical analysis of the sections from 21 subjects (lean 6; overweight 7; obese 8) that were stained with the respective antibodies. Staining intensity of TLR2 and TLR4 expression was greater (P < 0.05) in obese subjects compared to lean or overweight subjects (Figure 3A, B, D and E). It was noticed that staining intensities of TLR2 and TLR4 were observed in the areas of the adipose tissue infiltrated with inflammatory cells. TLR2 and TLR4 positive cells were more abundant in adipose tissue section from obese as compared to lean individuals (Figure 3A and D). Linear regression analysis also revealed a significantly positive correlation between TLR2/TLR4 expression levels and BMI (TLR2: r = 0.68, P = 0.0007; TLR4: r = 0.43, P = 0.052) (Figure 3C and F). We also measured the expression of TLRs’ adaptor proteins MyD88, IRAK1 and TRAF6 in adipose tissues used in the previous experiment. We found that only MyD88 levels were increased in adipose tissues from obese individuals (Figure 4A, B). Linear regression analysis revealed a significantly positive correlation between MyD88 protein expression and BMI (r = 0.58, P = 0.005) (Figure 4C).

### Increased expression of TNF- and IL-6 is correlated with TLRs expression in PBMCs of obese subjects

**Figure 5:**
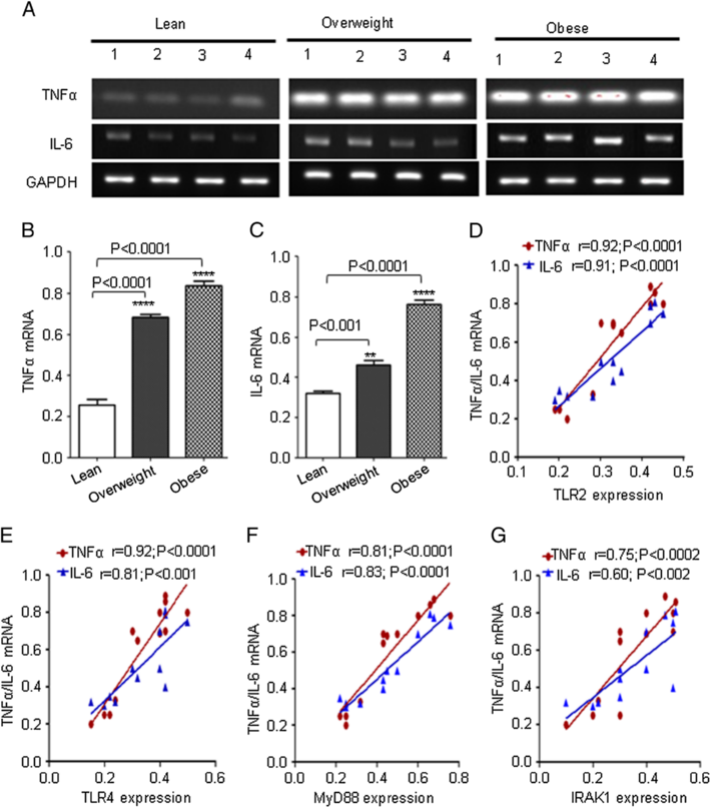
**Expression of IL**-**6 and TNF**-**α in PBMCs. **The PBMCs from 4 individuals in each group (lean, overweight and obese) were used for total RNA isolation. IL-6 and TNF-α mRNA were measured by RT-PCR (**A**). The RNA expression was normalized to GAPDH housekeeping gene expression. Cytokines expression is shown in arbitrary units for each group. Results are expressed relative to the expression level in controls and are represented as means ± SE values (**B **and **C**). A strong association of TLR2/ TLR4 mRNA with cytokines was observed in PBMCs (**D **and **E**). A simple linear regression analysis indicated a significant positive association between adopter protein (MyD88 or IRAK1) and cytokines (**F **and **G**).

It has been known that elevated circulating levels of TNF-α and IL-6 in obese subjects play an important role in the development of insulin resistance [19-22]. However, less information is available about the simultaneous expression of TNF-α and IL-6 with TLRs in PBMCs from same individuals. Therefore, we investigated the expression of these cytokines in the PBMCs and their association with the expression of TLRs and their adaptor proteins. Transcript levels of TNF-α and IL-6 were significantly higher in PBMCs from overweight and obese subjects compared to lean subjects (P < 0 · 05) (Figure 5A, B, C). Linear regression analysis demonstrated an association between the transcript expression levels of cytokines and TLRs expression (TLR2 vs TNF-α: r = 0.92, P < 0.0001; TLR2 vs IL-6: r = 0.91, P < 0.0001; TLR4 vs TNF-α: r = 0.92, P < 0.0001; TLR4 vs IL-6: r = 0.81, P < 0.001) (Figure 5D, E). The increased expression of TNF-α and IL-6 in PBMCs from obese subjects correlated with increased expression of TLRs’ adaptor proteins (MyD88 vs TNF-α: r = 0.9, P < 0.0001; MyD88 vs IL-6: r = 0.91, P < 0.0001; IRAK1 vs TNF-α: r = 0.86, P < 0.0002; IRAK1 vs IL-6: r = 0.77, P < 0.002) (Figure 5F, G).

### Expression of TLRs in PBMCs from obese subjects with type 2 diabetes

**Figure 6:**
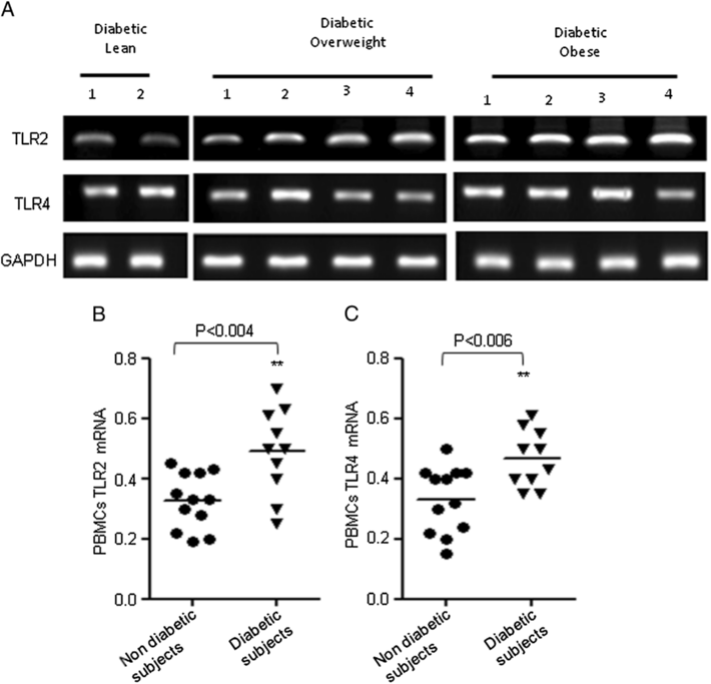
**Expression of TLRs in subjects with type 2 diabetes. **PBMCs from 10 individuals with diabetes (lean: 2; overweight: 4; obese: 4) were used for total RNA isolation. TLR2/TLR4 mRNA was measured by RT-PCR (**A**). The RNA expression was normalized to GAPDH housekeeping gene expression. TLRs’ expression is shown in arbitrary units for each group. Results are expressed relative to the expression level in controls and horizontal bars show the mean of each group. The values of expression of TLRs in PBMCs from 12 non diabetic subjects were taken from Figure 1 (**B **and **C**).

Since our data showed that TLRs expression was high in obese individuals and was significantly correlated with inflammatory cytokines which causes insulin resistance. Therefore, we measured TLRs’ expression in PBMCs from 10 diabetic subjects with different BMI. Our results showed that the subjects with type-2 diabetes had significantly elevated mRNA levels of TLR2 and TLR4 as compared with non-diabetic obese subjects (Figure 6A, B and Figure 1A). The basal levels of TNF-α and IL-6 were high in all groups of diabetic subjects and there was no significance difference (data not shown).

### Increased TLRs expression is correlated with FBG and HbAIc

Spearman’s rank correlation was performed to assess the association of TLRs’ expression with FBG and HbAIc. Correlation analysis showed that elevated levels of TLR2 in obesity was significantly correlated with FBG (r = 0.61; P < 0.0025) and HbAIc (r = 0.44; P < 0.03) (Table 2). Similarly, TLR4 positively correlated with FBG (r = 0.52; P < 0.01) and HbAIc (r = 0.48; P < 0.03) (Table 2).

**Table 2:** Correlation of TLRs with Fasting Blood Glucose and HbA1c

**TLRs**	**Metabolic parameters**	**Spearman r**	**P**
TLR2	Fasting Blood Glucose (mmol/l)	0.6107	0.0025**
	HbA1c	0.442	0.039*
TLR4	Fasting Blood Glucose (mmol/l)	0.52	0.01*
	HbA1c	0.48	0.03*

## Discussion

Obesity is associated with a low-grade systemic chronic inflammatory state characterized by abnormal production of pro- and anti-inflammatory adipocytokines leading to immune dysfunction and contributing to increased disease risk. Precise triggers for obesity-induced inflammation are not yet fully understood. Activation of PBMCs is an important initial step in the cascades of events leading to many inflammatory diseases including insulin resistance. Since expression of receptors on cells is a key element in the regulation of proinflammatory cytokines, we determined whether TLRs (TLR2 and TLR4) and inflammatory cytokines expression was simultaneously modulated on PBMCs in obesity.

Our data demonstrated alterations in TLR2 and TLR4 expressions in PBMCs and adipose tissues from obese subjects. In parallel, abnormalities in cytokines expression were found in PBMCs from obese individuals. Moreover, we found that there was a strong association between TLRs’ expression and cytokines (IL-6 and TNF-) measured simultaneously in PBMCs. Recent findings indicate that the TLRs which are up-regulated in the affected tissue of most inflammatory disorders can mediate crosstalk between the immune systems and body metabolism [23]. Increased TLRs activity was reported in patients with metabolic syndrome [24]. Elevated TLR2 and TLR4 expression was assessed in artherosclerotic lesions [25]. Higher expression of TLRs 3 and 4 in other conditions, such as an early stage of RA suggests that modulation in TLR expression does result during inflammatory states [26]. We found that markedly increased TLR2 and TLR4 levels in adipose tissue from obese subjects related with their increased expression on PBMCs. This could be the result of migration of inflammatory monocytes/macrophages from the peripheral compartment to the adipose tissue. Shi et al. (2006) argued that the increased expression of TLR4 mRNA in total adipose tissue extracts in two models of obesity could be due, in part, to increased numbers of macrophages known to reside in fat tissue of obese animals. Since a preferential macrophage infiltration into obese adipose tissue was demonstrated [27], it was suggested that the toll-like receptors’ expression in adipose tissue was mainly due to macrophages [28].

The enhanced expression of cytokines we observed in obese subjects could be explained by an elevated expression of TLRs on PBMCs. Other factors, such as increased free fatty acids, can influence TLRs responsiveness and explain the increased response observed in obese individuals. Saturated free fatty acids which are elevated in case of obesity are able to augment TLR induced cytokine production [29,30]. This could be a reason, in part, that obese individuals are more prone to developing insulin resistance. There is a clear link between TLR activation and insulin resistance. Free fatty acids cause insulin resistance in TLRs dependent manner. Recently pro-inflammatory effects of resistin were seen through its activation of TLR4 [31] and other endogenous ligands such as HMGB1 or hyaluronan fragments or HSPs also served as TLR4 or TLR2 activators [32]. Mice lacking TLR2 are substantially protected from diet-induced adiposity, insulin resistance, hypercholesterolemia, and hepatic steatosis and TLR2 deletion was associated with attenuation of adipocyte hypertrophy as well as diminished macrophage infiltration and inflammatory cytokine expression [33]. It was reported that the absence of TLR2 attenuated local inflammatory cytokine expression and related signaling and increased insulin action specifically in the liver [34]. Notably, only a few studies so far have described the responsiveness of TLRs to free fatty acid in insulin resistance and cytokine production. Interestingly, TLR expression modulation on PBMCs and adipose tissues in obese individuals remains poorly defined. The present data show over-expression of TLR2 and TLR4 on both PBMCs and adipose tissues which may explain their increased response to endogenous TLR ligands. Our findings suggest that TLRs modulation is linked with expression of proinflammatory cytokines in obese individuals.

The prior data demonstrate a correlation between TLR2/4 expression and BMI in subjects with type 2 diabetes [30]; however, the exact mechanism by which these two clinical predictors are interlinked remains undefined. There is lacking information about the relation of TLRs modulation and BMI in obese individuals. Expression of TLR2 and TLR4 on PBMCs varied widely among the individuals with different BMI that we observed. The extent of the obesity-induced up-regulation of TLR2/TLR4 genes and related proinflammatory cytokines cascades related to the BMI values. We found that TLR2 and TLR4 and inflammatory cytokines were overexpressed in PBMCs from obese subjects and this TLRs/cytokines overexpression correlated with the BMI. Increased expression of TLRs was correlated with FBG and HbAIc. More importantly, our data show a remarkable difference in the level of expression of toll-like receptors between obese individuals with and without diabetes. Overall, these results suggest that the overexpression of TLR2 and TLR4 on both PBMCs and adipose tissues together with the enhanced production of proinflammatory cytokines may pave way for the development of insulin resistance in obese individuals, leading to type 2 diabetes.

In conclusion, we found that TLR2 and TLR4 were overexpressed on PBMCs/adipose tissues from obese subjects which correlated with the increased expression of proinflammatory cytokines. This association may explain a potential pathophysiological link between obesity and inflammation, to subsequently result in development of insulin resistance and type 2 diabetes.

## Competing interests

The authors declare that they have no competing interests.

## Authors’ contributions

RA conceived, designed the experiments, analyzed the data, wrote and edited the manuscript. AA performed most part of the experiments and analyzed the data. VA performed immunohistochemistry related experiments. AA carried out the biochemical profile of the study subject. FA recruited the study subjects and obtained clinical data of the subjects. MA and AB commented the article. SD coordinated the recruitment of the study subjects as an obesity research program coordinator/head. KB critically revised and commented the article. All authors read and approved the final version of the manuscript.
